# 3-Weeks of Exercise Training Increases Ischemic-Tolerance in Hearts From High-Fat Diet Fed Mice

**DOI:** 10.3389/fphys.2019.01274

**Published:** 2019-10-02

**Authors:** Neoma T. Boardman, Line Rossvoll, Jim Lund, Anne D. Hafstad, Ellen Aasum

**Affiliations:** Cardiovascular Research Group, Department of Medical Biology, Faculty of Health Sciences, UiT – The Arctic University of Norway, Tromsø, Norway

**Keywords:** obesity, exercise, mice, Langendorff, mitochondria, MVO_2_, infarct size, functional recovery

## Abstract

Physical activity is an efficient strategy to delay development of obesity and insulin resistance, and thus the progression of obesity/diabetes-related cardiomyopathy. In support of this, experimental studies using animal models of obesity show that chronic exercise prevents the development of obesity-induced cardiac dysfunction (cardiomyopathy). Whether exercise also improves the tolerance to ischemia-reperfusion in these models is less clear, and may depend on the type of exercise procedure as well as time of initiation. We have previously shown a reduction in ischemic-injury in diet-induced obese mice, when the exercise was started prior to the development of cardiac dysfunction in this model. In the present study, we aimed to explore the effect of exercise on ischemic-tolerance when exercise was initiated after the development obesity-mediated. Male C57BL/6J mice were fed a high-fat diet (HFD) for 20–22 weeks, where they were subjected to high-intensity interval training (HIT) during the last 3 weeks of the feeding period. Sedentary HFD fed and chow fed mice served as controls. Left-ventricular (LV) post-ischemic functional recovery and infarct size were measured in isolated perfused hearts. We also assessed the effect of 3-week HIT on mitochondrial function and myocardial oxygen consumption (MVO_2_). Sedentary HFD fed mice developed marked obesity and insulin resistance, and demonstrated reduced post-ischemic cardiac functional recovery and increased infarct size. Three weeks of HIT did not induce cardiac hypertrophy and only had a mild effect on obesity and insulin resistance. Despite this, HIT improved post-ischemic LV functional recovery and reduced infarct size. This increase in ischemic-tolerance was accompanied by an improved mitochondrial function as well as reduced MVO_2_. The present study highlights the beneficial effects of exercise training with regard to improving the ischemic-tolerance in hearts with cardiomyopathy following obesity and insulin resistance. This study also emphasizes the exercise-induced improvement of cardiac energetics and mitochondrial function in obesity/diabetes.

## Introduction

Cardiovascular disease is a major cause of morbidity and mortality in type 2 diabetic patients (Kannel and McGee, 1979). Obese and diabetic patients are also at risk of developing a specific cardiomyopathy. The pathogenesis of this cardiomyopathy is multifactorial and complex, including fibrosis, inflammation, mitochondrial dysfunction, altered substrate utilization, oxidative stress and altered Ca^2+^ handling (Bugger and Abel, 2014; Jia et al., 2018). An early hallmark is also elevated myocardial oxygen consumption (MVO_2_), leading to cardiac inefficiency (Boudina et al., 2007; Wright et al., 2009; Cole et al., 2011; Hafstad et al., 2013; Lund et al., 2015). Diabetes also increases the risk of acute myocardial infarction as well as death following infarction (Haffner et al., 1998). In accordance, experimental studies using animal models of obesity, insulin resistance and/or diabetes generally show less tolerance to ischemic-reperfusion injury. Although the pathophysiological mechanisms contributing to ischemic injury in normal hearts has been subject to a comprehensive investigation, the underlying mechanisms leading to the higher ischemic susceptibility in the diabetic heart are not well known. As increased oxygen consumption will be particularly disadvantageous under conditions of limited oxygen availability, there is reason to suggest that the obesity/diabetes-induced increase in MVO_2_ contributes to a higher susceptibility to ischemic-reperfusion injury.

Exercise training is considered a key element in the management of type 2 diabetes (Colberg et al., 2010; Zanuso et al., 2010) as well as in the prevention and treatment of cardiovascular diseases (Myers et al., 2002). In support of this, experimental studies have shown that chronic exercise not only reduces obesity and insulin resistance (Zanuso et al., 2010) but also prevents or ameliorates the development of cardiac dysfunction (Bidasee et al., 2008; Lu et al., 2017). Accordingly, we have previously found reduced obesity and insulin resistance, accompanied by preserved cardiac function and decreased infarct size after 10 weeks of high-intensity exercise training (HIT) in mice fed a high-fat diet (HFD) for 18 weeks (Lund et al., 2015). As the exercise procedure was initiated prior to the development of cardiac dysfunction, its effect on the heart could have been due to delayed progression and severity of obesity/diabetes-related cardiomyopathy. In the present study, we aimed to explore the cardiac effect of exercise training with regard to improving ischemic-tolerance in a model with obesity-mediated cardiomyopathy.

## Materials and Methods

### Animals and Exercise Protocol

C57BL/6J male mice (5–6 weeks) were purchased from Charles River Laboratories (Germany). Obesity and insulin resistance were induced by feeding the mice a (HFD 58V8, TestDiet, United Kingdom, 60% of calories from fat) for 20–22 weeks. All mice received chow and drinking water *ad libitum* and were housed at 23°C on a reversed light-dark cycle. Due to the nocturnal nature of mice, all exercise training occurred during their dark cycle. During the last 3 weeks of the feeding regime, the mice were assigned to maintain a sedentary lifestyle (HFD_SED_) or high-intensity interval training (HFD_HIT_) by treadmill running 5 days/week as previously described (Hafstad et al., 2011, 2013). The exercise protocol consisted of 10 bouts of 4-min, high-intensity, treadmill running at 25° inclination, corresponding to 85–90% of VO_2__*max*_, interspersed by 2 min active rest (Hafstad et al., 2011, 2013). Aerobic capacity, determined as VO_2__*max*_, was assessed before and after the 3–wk exercise protocol, using a treadmill in a metabolic chamber (Columbus Instruments, Columbus, OH) (Hafstad et al., 2011). Blood was collected from the saphenous vein following a 4-h fasting period. Blood glucose concentration was measured with a glucometer (FreeStyle Lite, Alameda, CA, United States), and plasma insulin was analyzed using commercial kits from DRG Diagnostics (Marburg, Germany). Animal experiments were approved by the Norwegian National Animal Research Authority (FDU ID 3698), which conforms to the National Institute of Health guidelines (NIH publication No. 85-23, revised 1996) and European Directive 2010/63/EU.

### Assessment of Left Ventricular Susceptibility to Ischemic Injury

Isolated perfused hearts were subjected to ischemia-reperfusion, and post-ischemic recovery of left ventricular (LV) function was assessed using an intra-ventricular fluid-filled balloon where a vent was also inserted into the LV, through the apex. The volume of the balloon was adjusted to give an end-diastolic pressure of 5–10 mmHg. The hearts were perfused in a recirculating mode, with a modified Krebs-Henseleit bicarbonate buffer supplemented with 5 mM glucose and 0.4 mM palmitate prebound to 3% BSA. After 20 min stabilization and 25 min global ischemia, post-ischemic functional recovery was measured over a 60 min period. Reperfusion was continued for an additional 40 min to allow for determination of infarcted tissue. At the end of reperfusion, hearts were frozen at −20°C, prior to slicing and staining using a 1% 2,3,5-triphenyl-2H-tetrazolium chloride solution. Infarct size was determined using ImageJ software (National Institutes of Health, Bethesda, MD, United States). Fiber-optic oxygen probes (FOXY-AL300; Ocean Optics, Duiven, Netherlands) were used to assess PO_2_ in the perfusion buffer above the aortic cannula and in the pulmonary artery. Coronary flow and the arterial-venous difference of PO_2_ was used to determine myocardial oxygen consumption (MVO_2_) as previously described (Lund et al., 2015; Boardman et al., 2017). In order to assess a work-independent (unloaded) MVO_2_, the hearts were subjected to an unloaded perfusion condition to minimize the workload (Boardman et al., 2009).

### Mitochondrial Respiration

Cardiac mitochondria were isolated from hearts harvested prior to the ischemic insult. Briefly, tissue from the left ventricle was homogenized and trypsinized (5 mg/mL) in isolation buffer (250 mM sucrose, 0.5 mM EDTA, 10 mM Tris; pH 7.4). After a further homogenization, and differential centrifugation, mitochondrial pellets were suspended in respiration buffer containing 0.5 mM EGTA, 3 mM MgCl_2_, 60 mM K-lactobionate, 20 mM taurine, 10 mM KH_2_PO_4_, 20 mM HEPES, 110 mM sucrose, 1 g/L BSA, 280 U/mL catalase, 20 mM histidine, 20 μM vitamin E succinate, 3 mM glutathione, 1 μM leupeptin, 2 mM glutamate, 2 mM malate, and 2 mM ATP; pH 7.1. Mitochondria were kept on ice for 1 h prior to mitochondrial respiration experiments. Oxygen consumption was measured using an oxygraph (O2-k, Oroboros Instruments, Austria), where pyruvate (5 mM) and malate (2 mM) or palmitoyl-CoA (25 μM), L-carnitine (5 mM), and malate (2 mM) served as substrates. V_0_ was defined as the respiration in the presence of substrates before ADP was added. An oxidative phosphorylation state (V_*max*_) was defined as the respiration peak after adding 100 μmol/L ADP. Respiration rates were adjusted to protein content (Bradford Protein Assay Kit). The respiratory coupling ratio (RCR) was calculated as V_*max*_/V_0_.

### Statistical Analysis

All data are presented as mean ± standard error of means. Numbers of observations are presented as “n.” Differences between three groups were analyzed using one-way ANOVA with multiple comparisons (Holm-Sidak method as *post hoc* test). Where the normality test failed (Shapiro–Wilk test), a Mann–Whitney rank-sum test was performed.

## Results

### The Effect of HFD and 3-Week HIT on Obesity, Insulin Resistance and Aerobic Capacity

Sedentary mice fed an obesogenic diet for 20–22 weeks (HFD_SED_) developed obesity as indicated by elevated bodyweight and perirenal fat mass when compared to chow fed control (CON) mice (Table 1). Diet-induced obesity was accompanied by a marked insulin resistance (HOMA-IR), as well as reduced aerobic capacity (VO_2__*max*_).

**TABLE 1:** Animal characteristics of control mice (CON) and HFD-fed obese mice subjected to 3 weeks of high-intensity (HFD_HIT_) exercise training or a sedentary (HFD_SED_) lifestyle.

	**CON**	**HFD_SED_**	**HFD_HIT_**
*N*	20	20	13
Body weight (g)	31.5 ± 0.6^∗^	47.6 ± 0.7	43.2 ± 1.2^∗^
Tibia length (mm)	18.2 ± 0.1	18.2 ± 0.1	18.2 ± 0.1
Perirenal fat mass (g)	0.3 ± 0.1^∗^	1.5 ± 0.1	1.3 ± 0.1^∗^
Blood glucose _fasted_ (mmol/L)	6.3 ± 0.3	7.5 ± 0.2	6.0 ± 0.2^∗^
Plasma insulin _fasted_ (μg/L)	0.8 ± 0.3^∗^	3.2 ± 0.3	2.3 ± 0.4
HOMA-IR	5.5 ± 0.1^∗^	26.6 ± 2.6	15.2 ± 2.6^∗^
VO_2__max_ (mL/min/kg)	48.8 ± 0.7^∗^	43.2 ± 0.5	47.4 ± 0.3^∗^
Heart weight (mg wwt)	143 ± 4	150 ± 3	148 ± 3
Heart weight/tibia length	7.81 ± 0.32	8.12 ± 0.25	8.26 ± 0.17

*Data are means ± SE. Aerobic capacity(VO_2__*max*_) was measured in 10 and 8 animals, respectively. Homeostatic model assessment of insulin resistance; HOMA-IR. ^∗^*p* < 0.05 vs. HFD_*SED*_.*

As expected, subjecting obese mice to 3-week HIT (HFD_HIT_) resulted in increased aerobic capacity and a small but significant lowering of body weight and perirenal fat mass (Table 1). HIT also reduced insulin resistance (HOMA-IR), mainly due to a reduction in circulating insulin levels. It should be noted that 3-week of HIT did not induce cardiac hypertrophy, which was supported by unaltered gene expression of hypertrophic markers (data not shown).

### The Effect of HFD and 3-Week HIT on Post-ischemic Functional Recovery and Infarct Size

Previous reports from our group, have demonstrated delayed LV relaxation (increased Tau), increased end-diastolic pressure and an elevated end-diastolic pressure-volume relationship (Pedersen et al., 2018) in this mouse model following LV pressure-volume analysis. In the present study, intraventricular pressure was measured using a fluid-filled balloon in Langendorff perfused hearts (Table 2). However, this perfusion mode does not allow for the reliable determination of diastolic function (Pedersen et al., 2018). Although this represents a clear limitation of the present study, it should be noted that we have, in this model, repeatedly documented a diastolic dysfunction at identical age/feeding duration that is relevant for this study (18–20 week on obesogenic diets).

**TABLE 2:** Steady-state parameters of LV function obtained in isolated Langendorff perfused hearts from control mice (CON) and HFD-fed obese mice subjected to 3 weeks of high-intensity exercise training (HFD_HIT_) or a sedentary lifestyle (HFD_SED_).

	**CON**	**HFD_SED_**	**HFD_HIT_**
*N*	13	16	16
Coronary flow (mL/min)	3.2 ± 0.1	3.7 ± 0.2	3.6 ± 0.2
Heart rate (bpm)	315 ± 10	307 ± 9	293 ± 9
LV max-systolic pressure (mmHg)	137 ± 8	151 ± 10	168 ± 12
LV end-diastolic pressure (mmHg)	9.4 ± 0.7	9.9 ± 0.9	10.2 ± 0.5
LV developed pressure (mmHg)	128 ± 8	141 ± 11	158 ± 11
dP/dt_max_ (mmHg/sec)	4624 ± 384	5603 ± 407	6559 ± 376
dP/dt_min_ (mmHg/sec)	−3394 ± 177	−3818 ± 281	−4339 ± 203
RPP (mmHg^∗^bpm)	40163 ± 2536	43218 ± 3430	45850 ± 2929

**FIGURE 1:**
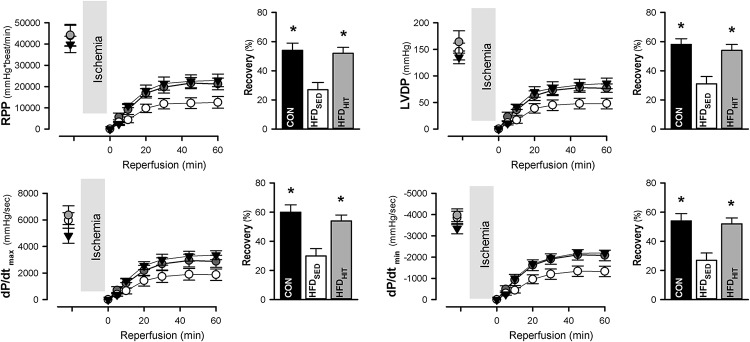
Rate-pressure product (RPP), LV developed pressure (LVDP), and the maximum and minimum of the pressure derivative (dP/dt_*max*_, dP/dt_*min*_) in isolated perfused hearts from control mice (CON, *n* = 8) and HFD fed mice subjected to a sedentary lifestyle (HFD_SED_, *n* = 10) or to 3 weeks of high-intensity exercise training (HFD_HIT_, *n* = 6). Bars indicate the calculated post-ischemic recovery (% of pre-ischemic values) of these parameters. Data are means ± SE. ^∗^*p* < 0.05 vs. HFD_SED_.

*Data are means ± SE. These data include left ventricular (LV) functional assessment in hearts subjected to ischemia reperfusion (included in Figure 1) and hearts used for isolation of mitochondria (included inFigure 3). dP/dt_*max*_ and dP/dt_*min*_; maximum positive and negative first-time derivative of LV pressure. RPP; rate-pressure-product (developed pressure × heart rate). End-diastolic pressure was adjusted to be between 5 and 10 mmHg. ^∗^*p* < 0.05 vs. HFD_*SED*_.*

The Langendorff perfusion mode is well suited to examine changes in tolerance to ischemia-reperfusion. Functional recovery was followed during the first 60 min of reperfusion after a period of global ischemia. Hearts from HFD_SED_ mice showed impaired recovery of the rate-pressure-product (RPP, given as % of the pre-ischemic value) when compared to hearts from CON mice (Figure 1). As heart rate was not different between the groups, the reduced recovery of RPP was primarily due to the reduced recovery of LV developed pressure (Figure 1). Likewise, we also found a significantly impaired recovery of both the maximum and minimum of the pressure derivative (dP/dt_*max*_ and dP/dt_*min*_) indicating increased post-ischemic stunning in HFD_SED_ hearts (Figure 1). Three weeks of exercise training was found to significantly improve functional recovery not only of RPP, but also LV developed pressure, as well as dP/dt_*max*_ and dP/dt_*min*_ (Figure 1). HR was not altered by exercise, which emphasized that exercise increased LV contraction by improving both contractility and relaxation.

**FIGURE 2:**
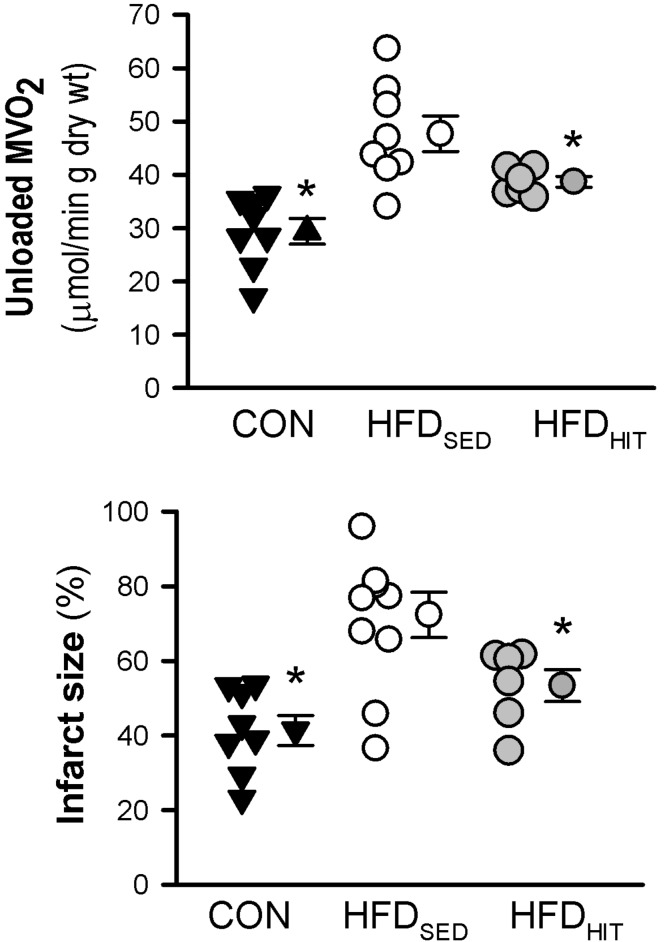
Unloaded MVO_2_ and infarct size was measured in isolated perfused hearts from control mice (CON, *n* = 8) and HFD fed mice subjected to a sedentary lifestyle (HFD_SED_, *n* = 10) or 3 weeks of high-intensity exercise training (HFD_HIT_, *n* = 6). The unloaded MVO_2_ was obtained by deflating the balloon in the LV for a short period during baseline conditions and pacing the hearts at 7 Hz. Data are means ± SE. ^∗^*p* < 0.05 vs. HFD_SED_.

Impaired functional recovery of post-ischemic function in HFD_SED_ hearts was accompanied by increased infarct size when compared to CON (Figure 2). In addition, 3-week HIT was found to reduce ischemia-reperfusion induced cell death (i.e., infarct size) in hearts from HFD mice.

### The Effect of HFD and 3-Week HIT on MVO_2_ and Mitochondrial Respiration

**FIGURE 3:**
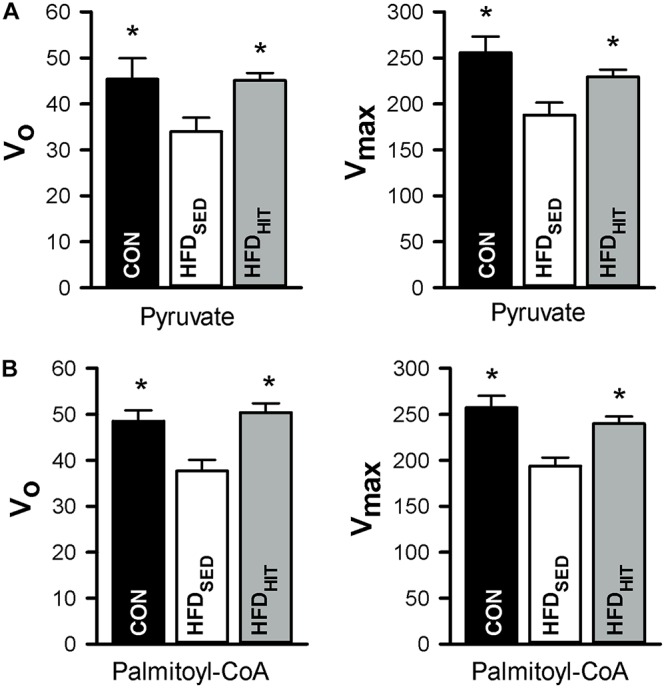
Respiration in isolated mitochondria (nmol O_2_/min/mg protein) from hearts of control mice (CON, *n* = 5) mice and HFD-fed obese mice subjected to a sedentary lifestyle (HFD_SED_, *n* = 7) or 3 weeks of high-intensity exercise training (HFD_HIT_, *n* = 7). The respiratory medium contained pyruvate (5 mM) and malate (2 mM) **(A,B)** or palmitoyl-CoA (25 μM), L-carnitine (5 mM) and malate (2 mM). V_0_ respiration, the respiratory state before ADP is added. V_*max*__,_ the respiration peak after adding 100 μmol/L ADP. Data are means ± SE. ^∗^*p* < 0.05 vs. HFD_SED_.

This study confirmed that HFD_SED_ hearts demonstrate higher oxygen consumption (MVO_2_) compared to CON hearts when perfused in an unloaded state (Lund et al., 2015). Further, we examined mitochondrial respiration in isolated mitochondria from hearts perfused for 30 min, but not subjected to ischemia-reperfusion. The ADP-dependent oxidative phosphorylation state (V_*max*_) in mitochondria from HFD_SED_ hearts was reduced when compared to CON hearts, both when using pyruvate as well as palmitoyl-CoA as respiratory substrates (Figure 3). As ADP-independent respiration (V_0_) was also lower in these hearts, the rate-dependent RCR remained the same in CON and HFD_SED_ (pyruvate: 5.7 ± 0.3 and 5.6 ± 0.3, palmitoyl-CoA: 5.3 ± 0.1 and 5.2 ± 0.2, respectively).

We have previously demonstrated that 10 weeks HIT reduced unloaded MVO_2_ (Lund et al., 2015). The present study shows that 3 weeks of HIT was sufficient to induce this effect (Figure 2). In isolated cardiac mitochondria, 3-week HIT was also shown to increase oxidative phosphorylation (V_*max*_) under both substrate conditions (Figure 3). Interestingly, exercise also significantly increased V_0_, indicating an exercise-mediated induction of a mitochondrial proton-leak. Due to a concomitant increase in both the ADP-dependent and -independent O_2_ consumption, we did not find exercise to alter RCR (pyruvate: 5.1 ± 0.3, palmitoyl-CoA: 4.9 ± 0.2). Finally, although 10 weeks of HIT was previously shown to increase CS activity in cardiac tissue in normal hearts (Hafstad et al., 2013), 3-week HIT was not sufficient to alter CS activity in cardiac tissue from HFD mice (10.6 ± 1.0 vs. 12.3 ± 0.4 IU/g wwt, HFD_SED_ vs. HFD_HIT_, respectively).

## Discussion

We have previously shown in diet-induced obese mice, that exercise training initiated prior to changes in cardiac function, prevented the subsequent development of dysfunction (Hafstad et al., 2013; Lund et al., 2015) as well as exacerbated infarction (Lund et al., 2015). As this effect could have been due to delayed progression of the cardiomyopathy (because of reduced obesity and insulin resistance), we have now explored the effect of exercise on cardiac ischemic-tolerance in mice with obesity-mediated cardiomyopathy. We found that a shorter duration of exercise, initiated at the end of the feeding protocol, improved ischemic-tolerance, despite having only mild effects on co-morbidities. We also found that this effect was associated with an exercise-mediated reduction in myocardial oxygen consumption (MVO_2_) and improved cardiac mitochondrial respiration.

In accordance with previous studies (Lund et al., 2015; Pedersen et al., 2018), feeding C57BL/6J mice an HFD for more than 18 weeks led to obesity, insulin resistance and low aerobic capacity (VO_2__*max*_). These mice also develop impaired LV function, primarily manifested as a diastolic dysfunction (Lund et al., 2015; Pedersen et al., 2018). A hallmark of obesity/diabetes related cardiomyopathy is mechanical inefficiency (i.e., the ratio between cardiac work and MVO_2_) (Cole et al., 2011; Hafstad et al., 2013; Lund et al., 2015). Cardiac inefficiency occurs prior to development of dysfunction (Wright et al., 2009; Hafstad et al., 2013), and is due to a higher oxygen cost for non-mechanical processes (Hafstad et al., 2013; Lund et al., 2015). Accordingly, in the present study, hearts from the sedentary HFD fed mice, showed a higher unloaded MVO_2_. As a high O_2_ consumption is particularly detrimental under conditions of limited O_2_ availability, this O_2_ wasting can contribute to render these hearts more susceptible to an ischemic-injury. In support of this, the present study, as well as a range of other studies (Yi et al., 2011; Pons et al., 2013; Littlejohns et al., 2014; Lund et al., 2015), have reported a decreased tolerance to myocardial ischemia-reperfusion in models of obesity/diabetes.

Chronic exercise training improves obesity and insulin resistance (Colberg et al., 2010; Qiu et al., 2014), and may thus delay or ameliorate the severity of diabetic cardiomyopathy. In accordance, experimental studies from our lab have demonstrated that 10 weeks of exercise training of diet-induced obese mice delayed the progression of obesity, reduced insulin resistance (Hafstad et al., 2013; Lund et al., 2015), prevented the development of LV dysfunction (Hafstad et al., 2013; Lund et al., 2015) and decreased cardiac susceptibility to ischemic-injury (Lund et al., 2015). Notably, in these studies, exercise was started prior to the development of dysfunction (i.e., halfway through the 18-week feeding protocol). The cardiac effects could therefore be due to a postponed development of cardiomyopathy. In the present study, we wanted to examine the effect of shorter exercise period, which did not prevent the development of cardiomyopathy. Therefore, mice were fed HFD for 20–22 weeks, and subjected to HIT during the final 3 weeks of the feeding period.

Compared to the longer exercise protocol (10-week HIT) (Lund et al., 2015), the systemic effects of 3-week HIT were, as expected, less marked. Accordingly, while 10-week HIT reduced perirenal fat by approximately 36–44% (Lund et al., 2015; Boardman et al., 2017), the corresponding reduction by 3-week HIT was only 11%. Similarly, 3-week HIT also resulted in a lesser decrease in HOMA (30 vs. 60%), and a smaller increase in VO_2__*max*_ (7 vs. 20%). Finally, in contrast to 10-week, 3-week HIT was not sufficient to develop cardiac hypertrophy.

We have assessed ischemic susceptibility in hearts perfused in Langendorff perfusion mode with an intraventricular balloon. As this perfusion mode cannot detect changes in diastolic function in mouse hearts (Lund et al., 2015; Pedersen et al., 2018), the present study could not confirm the diastolic dysfunction that has been shown to develop in this model (Lund et al., 2015; Pedersen et al., 2018). Nor can the present study determine whether or not 3-week HIT ameliorated LV diastolic dysfunction. This study, however, clearly demonstrated that 3-week HIT improved post-ischemic functional recovery as well as reduced infarct size. Thus, despite a less marked modification of the associated co-morbidities, these hearts show improved ischemic-tolerance. This is also supported by Pons et al. (2013), who demonstrated that 4 weeks of exercise in *ob/ob* mice, reduced infarct size without altering hyperglycemia, hypercholesterolemia, hyperinsulinemia, fat mass or body weight.

While the mechanisms leading to exercise-induced cardioprotection in normal hearts have been widely studied, fewer studies have examined this in models of insulin resistance and/or type 2 diabetes. The present study supports that exercise can decrease myocardial O_2_ wasting found in diabetic cardiomyopathy. The increased O_2_-consumption and decreased efficiency are an early and consistent finding in these hearts (Wright et al., 2009; Hafstad et al., 2013). Although the mechanisms for O_2_ wasting are not fully understood, several of the reported pathophysiological changes in these hearts can impair mechanoenergetics, by altering ATP production and/or utilization. These changes include elevated fatty acid supply and/or utilization (Belke et al., 2000; Aasum et al., 2003), structural remodeling (i.e., myocardial stiffness) (How et al., 2006), impaired mitochondrial function and oxidative stress (Fauconnier et al., 2007; Anderson et al., 2009), as well as altered Ca^2+^ handling (Stolen et al., 2009; Epp et al., 2013). Importantly, several of these processes, can also be altered by exercise training (Hafstad et al., 2015). As a major part of the work-independent O_2_ consumption is linked to maintainance of Ca^2+^ homeostasis, exercise-mediated effects on myocardial Ca^2+^ transport (Stolen et al., 2009; Epp et al., 2013) can lead to decreased MVO_2_ (Hafstad et al., 2013). Exercise can also reduce oxidative stress [by increasing antioxidant capacity (Muthusamy et al., 2012)], and improve the redox state (Fisher-Wellman et al., 2013). As we found an exercise-induced mitochondrial proton leak [in the present study, and previously (Hafstad et al., 2013)], it could be suggested that obesity-mediated redox modification of the Ca^2+^ handling proteins is prevented by exercise due to leak-induced decrease in mitochondrial membrane potential.

## Conclusion

The present study highlights the beneficial effects of exercise training with regard to improving the ischemic-tolerance in hearts with obesity-induced cardiomyopathy. This study also emphasizes exercise-induced improvement of cardiac energetics and mitochondrial function.

## Data Availability Statement

The raw data supporting the conclusions of this manuscript will be made available by the corresponding author, without undue reservation, to any qualified researcher.

## Ethics Statement

This study was carried out in accordance with the recommendation of the National Institute of Health guidelines (NID publication No. 85–23, revised 1996) and European Directive 2010/63/EU. All protocols involving animals in the present study were approved by the Norwegian National Animal Research Authority.

## Author Contributions

NB, AH, and EA conceived and designed the research, and edited and revised the manuscript. NB, AH, JL, and LR performed the experiments. NB, LR, and EA analyzed the data. NB and EA interpreted the results of the experiments, prepared figures, and drafted the manuscript. NB, AH, JL, LR, and EA approved the final version of the manuscript.

## Conflict of Interest

The authors declare that the research was conducted in the absence of any commercial or financial relationships that could be construed as a potential conflict of interest.

## References

[B2] Aasum E. Hafstad A. D. Severson D. L. Larsen T. S. (2003). Age-dependent changes in metabolism, contractile function, and ischemic sensitivity in hearts from db/db mice. *Diabetes Metab. Res. Rev.* 52 434–441. 10.2337/diabetes.52.2.434 12540618

[B3] Anderson E. J. Kypson A. P. Rodriguez E. Anderson C. A. Lehr E. J. Neufer P. D. (2009). Substrate-specific derangements in mitochondrial metabolism and redox balance in the atrium of the type 2 diabetic human heart. *J. Am. Coll. Cardiol.* 54 1891–1898. 10.1016/j.jacc.2009.07.031 19892241 PMC2800130

[B4] Belke D. D. Larsen T. S. Gibbs E. M. Severson D. L. (2000). Altered metabolism causes cardiac dysfunction in perfused hearts from diabetic (db/db) mice. *Am. J. Physiol. Endocrinol. Metab.* 279 E1104–E1113. 10.1152/ajpendo.2000.279.5.E1104 11052966

[B5] Bidasee K. R. Zheng H. Shao C. H. Parbhu S. K. Rozanski G. J. Patel K. P. (2008). Exercise training initiated after the onset of diabetes preserves myocardial function: effects on expression of beta-adrenoceptors. *J. Appl. Physiol.* 105 907–914. 10.1152/japplphysiol.00103.2008 18583384 PMC2536823

[B6] Boardman N. Hafstad A. D. Larsen T. S. Severson D. L. Aasum E. (2009). Increased O2 cost of basal metabolism and excitation-contraction coupling in hearts from type 2 diabetic mice. *Am. J. Physiol. Heart Circ. Physiol.* 296 H1373–H1379. 10.1152/ajpheart.01264.2008 19286944

[B7] Boardman N. T. Hafstad A. D. Lund J. Rossvoll L. Aasum E. (2017). Exercise of obese mice induces cardioprotection and oxygen sparing in hearts exposed to high-fat load. *Am. J. Physiol. Heart Circ. Physiol.* 313 H1054–H1062. 10.1152/ajpheart.00382.2017 28801525

[B8] Boudina S. Sena S. Theobald H. Sheng X. Wright J. J. Hu X. X. (2007). Mitochondrial energetics in the heart in obesity-related diabetes: direct evidence for increased uncoupled respiration and activation of uncoupling proteins. *Diabetes Metab. Res. Rev.* 56 2457–2466. 10.2337/db07-0481 17623815

[B9] Bugger H. Abel E. D. (2014). Molecular mechanisms of diabetic cardiomyopathy. *Diabetologia* 57 660–671. 10.1007/s00125-014-3171-3176 24477973 PMC3969857

[B10] Colberg S. R. Sigal R. J. Fernhall B. Regensteiner J. G. Blissmer B. J. Rubin R. R. (2010). Exercise and type 2 diabetes: the american college of sports medicine and the american diabetes association: joint position statement executive summary. *Diabetes Care* 33 2692–2696. 10.2337/dc10-1548 21115771 PMC2992214

[B11] Cole M. A. Murray A. J. Cochlin L. E. Heather L. C. McAleese S. Knight N. S. (2011). A high fat diet increases mitochondrial fatty acid oxidation and uncoupling to decrease efficiency in rat heart. *Basic Res. Cardiol.* 106 447–457. 10.1007/s00395-011-0156-151 21318295 PMC3071466

[B12] Epp R. A. Susser S. E. Morissette M. P. Kehler D. S. Jassal D. S. Duhamel T. A. (2013). Exercise training prevents the development of cardiac dysfunction in the low-dose streptozotocin diabetic rats fed a high-fat diet. *Can. J. Physiol. Pharmacol.* 91 80–89. 10.1139/cjpp-2012-0294 23369057

[B13] Fauconnier J. Andersson D. C. Zhang S. J. Lanner J. T. Wibom R. Katz A. (2007). Effects of palmitate on Ca(2+) handling in adult control and ob/ob cardiomyocytes: impact of mitochondrial reactive oxygen species. *Diabetes Metab. Res. Rev.* 56 1136–1142. 10.2337/db06-0739 17229941

[B14] Fisher-Wellman K. H. Mattox T. A. Thayne K. Katunga L. A. La Favor J. D. Neufer P. D. (2013). Novel role for thioredoxin reductase-2 in mitochondrial redox adaptations to obesogenic diet and exercise in heart and skeletal muscle. *J. Physiol.* 591 3471–3486. 10.1113/jphysiol.2013.254193 23613536 PMC3731608

[B15] Haffner S. M. Lehto S. Ronnemaa T. Pyorala K. Laakso M. (1998). Mortality from coronary heart disease in subjects with type 2 diabetes and in nondiabetic subjects with and without prior myocardial infarction. *N. Engl. J. Med.* 339 229–234. 10.1056/nejm199807233390404 9673301

[B16] Hafstad A. D. Boardman N. Aasum E. (2015). How exercise may amend metabolic disturbances in diabetic cardiomyopathy. *Antioxid. Redox. Signal.* 22 1587–1605. 10.1089/ars.2015.6304 25738326 PMC4449627

[B17] Hafstad A. D. Boardman N. T. Lund J. Hagve M. Khalid A. M. Wisloff U. (2011). High intensity interval training alters substrate utilization and reduces oxygen consumption in the heart. *J. Appl. Physiol.* 111 1235–1241. 10.1152/japplphysiol.00594.2011 21836050

[B18] Hafstad A. D. Lund J. Hadler-Olsen E. Hoper A. C. Larsen T. S. Aasum E. (2013). High- and moderate-intensity training normalizes ventricular function and mechanoenergetics in mice with diet-induced obesity. *Diabetes Metab. Res. Rev.* 62 2287–2294. 10.2337/db12-1580 23493573 PMC3712042

[B19] How O. J. Aasum E. Severson D. L. Chan W. Y. Essop M. F. Larsen T. S. (2006). Increased myocardial oxygen consumption reduces cardiac efficiency in diabetic mice. *Diabetes Metab. Res. Rev.* 55 466–473. 10.2337/diabetes.55.02.06.db05-1164 16443782

[B20] Jia G. Hill M. A. Sowers J. R. (2018). Diabetic cardiomyopathy: an update of mechanisms contributing to this clinical entity. *Circ. Res.* 122 624–638. 10.1161/CIRCRESAHA.117.311586 29449364 PMC5819359

[B21] Kannel W. B. McGee D. L. (1979). Diabetes and cardiovascular disease. Framingham study. *JAMA* 241 2035–2038. 10.1001/jama.241.19.2035 430798

[B22] Littlejohns B. Pasdois P. Duggan S. Bond A. R. Heesom K. Jackson C. L. (2014). Hearts from mice fed a non-obesogenic high-fat diet exhibit changes in their oxidative state, calcium and mitochondria in parallel with increased susceptibility to reperfusion injury. *PLoS One* 9:e100579. 10.1371/journal.pone.0100579 24950187 PMC4065057

[B23] Lu G. Z. X. Sun Z. Shi X. Liu T. Xu X. (2017). Effects of exercise training on systolic and diastolic function of mice with diabetic cardiomyopathy. *Cardiol. Plus* 2 1–6. 10.4103/cp.cp_1_18

[B24] Lund J. Hafstad A. D. Boardman N. T. Rossvoll L. Rolim N. P. Ahmed M. S. (2015). Exercise training promotes cardioprotection through oxygen-sparing action in high fat-fed mice. *Am. J. Physiol. Heart Circ. Physiol.* 308 H823–H829. 10.1152/ajpheart.00734.2014 25637547

[B25] Muthusamy V. R. Kannan S. Sadhaasivam K. Gounder S. S. Davidson C. J. Boeheme C. (2012). Acute exercise stress activates Nrf2/ARE signaling and promotes antioxidant mechanisms in the myocardium. *Free Radic Biol. Med.* 52 366–376. 10.1016/j.freeradbiomed.2011.10.440 22051043 PMC3800165

[B26] Myers J. Prakash M. Froelicher V. Do D. Partington S. Atwood J. E. (2002). Exercise capacity and mortality among men referred for exercise testing. *N. Engl. J. Med.* 346 793–801. 10.1056/nejmoa011858 11893790

[B27] Pedersen T. M. Boardman N. T. Hafstad A. D. Aasum E. (2018). Isolated perfused working hearts provide valuable additional information during phenotypic assessment of the diabetic mouse heart. *PLoS One* 13:e0204843. 10.1371/journal.pone.0204843 30273374 PMC6166959

[B28] Pons S. Martin V. Portal L. Zini R. Morin D. Berdeaux A. (2013). Regular treadmill exercise restores cardioprotective signaling pathways in obese mice independently from improvement in associated co-morbidities. *J. Mol. Cell Cardiol.* 54 82–89. 10.1016/j.yjmcc.2012.11.010 23201226

[B29] Qiu S. Mintz J. D. Salet C. D. Han W. Giannis A. Chen F. (2014). Increasing muscle mass improves vascular function in obese (db/db) mice. *J. Am. Heart Assoc.* 3:e000854. 10.1161/JAHA.114.000854 24965025 PMC4309080

[B30] Stolen T. O. Hoydal M. A. Kemi O. J. Catalucci D. Ceci M. Aasum E. (2009). Interval training normalizes cardiomyocyte function, diastolic Ca2+ control, and SR Ca2+ release synchronicity in a mouse model of diabetic cardiomyopathy. *Circ. Res.* 105 527–536. 10.1161/CIRCRESAHA.109.199810 19679837

[B31] Wright J. J. Kim J. Buchanan J. Boudina S. Sena S. Bakirtzi K. (2009). Mechanisms for increased myocardial fatty acid utilization following short-term high-fat feeding. *Cardiovasc. Res.* 82 351–360. 10.1093/cvr/cvp017 19147655 PMC2675931

[B32] Yi W. Sun Y. Gao E. Wei X. Lau W. B. Zheng Q. (2011). Reduced cardioprotective action of adiponectin in high-fat diet-induced type II diabetic mice and its underlying mechanisms. *Antioxid. Redox. Signal.* 15 1779–1788. 10.1089/ars.2010.3722 21091073 PMC3159116

[B33] Zanuso S. Jimenez A. Pugliese G. Corigliano G. Balducci S. (2010). Exercise for the management of type 2 diabetes: a review of the evidence. *Acta Diabetol.* 47 15–22. 10.1007/s00592-009-0126-123 19495557

